# The Effectiveness of Nirsevimab on Antibiotic Use in Children Using Target Trial Emulation

**DOI:** 10.1093/cid/ciag406

**Published:** 2026-08-06

**Authors:** Mahaa M Ahmed, Torsten Joerger, Ziyi Wang, Yun Li, Jeffrey S Gerber

**Affiliations:** Clinical Futures, Children's Hospital of Philadelphia, Philadelphia, Pennsylvania, USA; Department of Pediatrics, Division of Infectious Diseases, University of Pennsylvania Perelman School of Medicine and Children's Hospital of Philadelphia, Philadelphia, Pennsylvania, USA; Clinical Futures, Children's Hospital of Philadelphia, Philadelphia, Pennsylvania, USA; Clinical Futures, Children's Hospital of Philadelphia, Philadelphia, Pennsylvania, USA; Department of Biostatistics, Epidemiology, and Informatics, University of Pennsylvania School of Medicine, Philadelphia, Pennsylvania, USA; Clinical Futures, Children's Hospital of Philadelphia, Philadelphia, Pennsylvania, USA; Department of Pediatrics, Division of Infectious Diseases, University of Pennsylvania Perelman School of Medicine and Children's Hospital of Philadelphia, Philadelphia, Pennsylvania, USA

**Keywords:** nirsevimab, RSV, vaccine, antibiotics, causal inference

## Abstract

**Background:**

Nirsevimab is a monoclonal antibody with demonstrated efficacy in preventing respiratory syncytial virus (RSV) infections in infants. Because acute respiratory tract infections (ARTIs), including viral infections, often lead to antibiotic prescribing, we aimed to determine the effectiveness of nirsevimab on antibiotic prescribing among infants with medically attended ARTIs.

**Methods:**

A primary care attendee cohort was established using electronic health record data from 32 diverse practices across a healthcare network to emulate a target trial comparing nirsevimab to no treatment among infants <8 months. Eligible infants were seen at a primary care practice within 14 days of life. Primary outcomes included time to first antibiotic prescription for (1) outpatient ARTIs, (2) outpatient encounters for bronchiolitis, and (3) RSV-related hospitalizations. Nirsevimab effectiveness was calculated as 1 minus the risk ratio for each primary outcome.

**Results:**

Among 15 341 patients who met inclusion criteria, 7413 received nirsevimab. Compared with no treatment, nirsevimab administration led to a 14.4% (95% CI: 7.8%, 20.6%) reduction in antibiotic prescribing for outpatient ARTIs, a 40.3% (95% CI: 25.1%, 52.5%) reduction in antibiotic prescribing for outpatient bronchiolitis, and a 69.4% (95% CI: 4.8%, 90.1%) reduction in antibiotic prescribing for RSV-related hospitalizations.

**Conclusions:**

Nirsevimab administration in primary care reduced antibiotic use for ARTIs in both ambulatory and inpatient settings.

## Teaser

This cohort study within a pediatric primary care network using target trial emulation including 15 341 patients found significant reductions in antibiotic prescribing for acute respiratory tract infections. Preventing RSV infections with nirsevimab has the secondary benefit of reducing antibiotic prescribing.

## Graphical Abstract

**Graphical Abstract:**
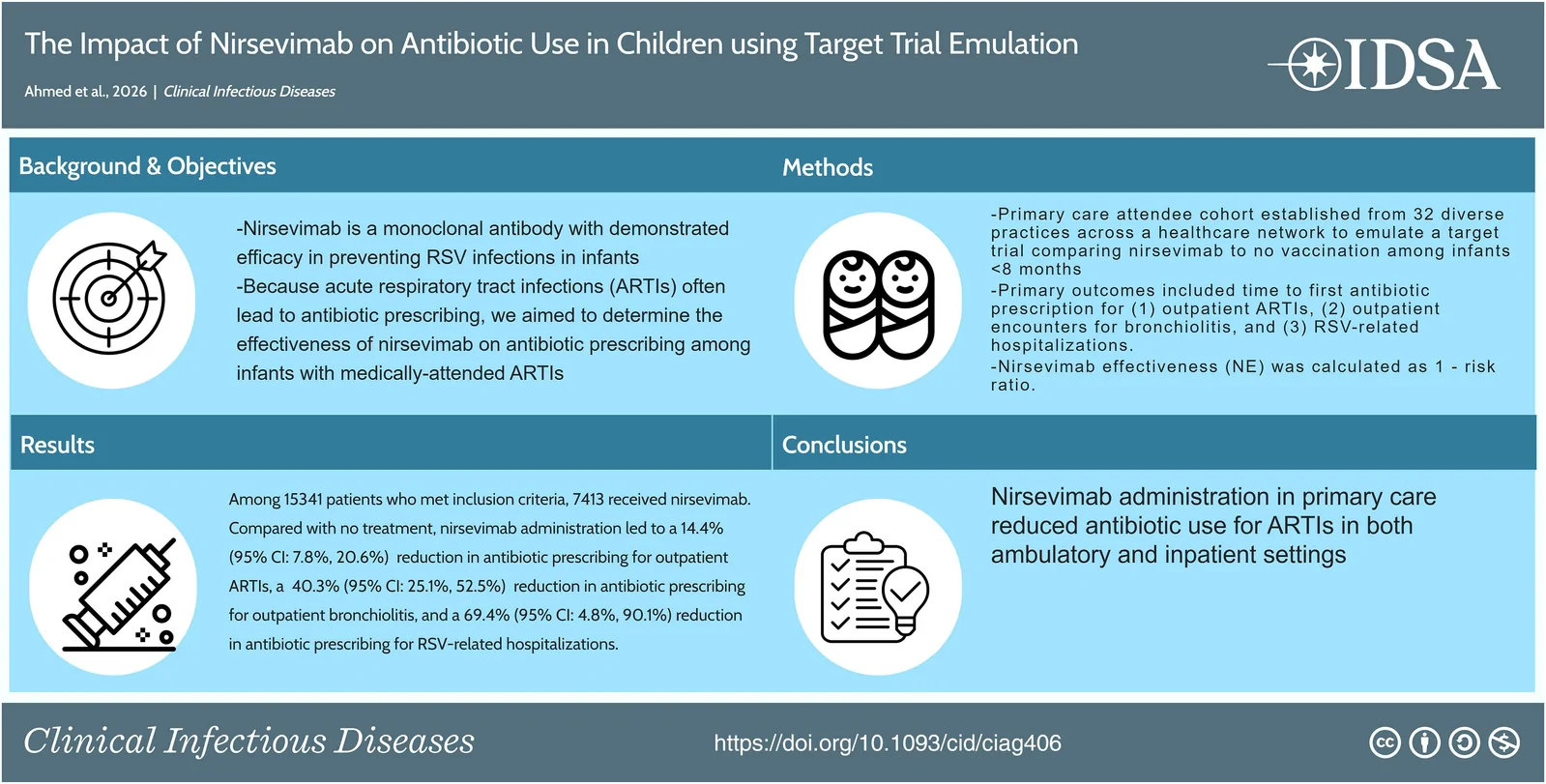
This graphical abstract is also available at Tidbit: https://tidbitapp.io/institutional-portal/clinical-infectious-diseases/tidbits/the-impact-of-nirsevimab-on-antibiotic-use-in-children-using-target-trial-emulation-bb1458ab-5040-436d-80a5-de43ff1c37c9/update

Respiratory syncytial virus (RSV) causes seasonal epidemics of respiratory infections in children, peaking between October and April each year in the United States [1]. The burden of these infections is substantial. Respiratory syncytial virus is estimated to be responsible for 1 out of every 3 hospitalizations for acute respiratory tract infections (ARTIs) in children less than 5 years of age, with children less than 6 months of age at the start of RSV season at highest risk for hospitalization [2]. In addition to 57 000 hospitalizations, it is estimated that RSV is responsible for more than 1.5 million visits to pediatrician offices each year in the United States [3].

Antibiotics are the most common class of medication prescribed to children under 2 years of age, with more than 10 million outpatient prescriptions in this age group in the United States each year, the majority for ARTIs [4, 5]. Approximately half of children hospitalized with RSV and up to 30% of children who present to an outpatient clinic with RSV receive antibiotics [6–8]. While beneficial for treating bacterial infections, antibiotic use is associated with a number of harmful effects, including antibiotic-associated diarrhea, adverse drug reactions, increased antimicrobial resistance, and alterations to a developing microbiome [9]. Vaccinations against bacterial pathogens, including *Haemophilus influenzae type b* and *Streptococcus pneumoniae*, as well as influenza, a respiratory virus, have been associated with decreases in subsequent antibiotic prescribing in children [10–12].

Nirsevimab is a long-acting monoclonal antibody designed for RSV prophylaxis recommended for infants younger than 8 months entering their first RSV season and high-risk infants 8–19 months old [13]. Nirsevimab has been shown in both clinical trials and postmarketing observational studies to prevent RSV-associated hospitalizations and medically attended outpatient visits in children [14, 15]. However, to date, clinical trials have not evaluated the efficacy of nirsevimab in reducing antibiotic prescriptions as a primary endpoint. Because (1) ARTIs are the most common indication for sick visits in infants, (2) antibiotic prescribing for viral ARTIs, including those caused by RSV, is common, and (3) RSV infections can either mimic or lead to bacterial illnesses (eg, acute otitis media and pneumonia), nirsevimab use might indirectly reduce safely avoidable antibiotic exposure in this population. Therefore, we used target trial emulation to determine the effectiveness of nirsevimab in reducing antibiotic use for ARTIs in both outpatient and inpatient settings among infants in their first RSV season between 2023 and 2025.

## METHODS

### Study Design and Setting

The Children's Hospital of Philadelphia (CHOP) institutional review board approved this study and granted a waiver of consent.

The CHOP Primary Care Network consists of 32 practices across Pennsylvania and New Jersey with more than 250 board-certified pediatricians and advanced registered nurse practitioners serving more than 300 000 children. More than 500 000 outpatient visits to these practices were recorded in 2023. Only 3 “academic practices” serve as residency program training sites, while the remaining 29 practices are community based. All 32 practices use a common electronic health record (EHR) EpiCare (Epic Systems, Inc.) for all encounter documentation and orders, including prescriptions, eligibility criteria, and treatment assignments.

The 2023–2024 RSV season was defined as 16 October 2023 through 31 March 2024. The 2024–2025 RSV season was defined as 3 October 2024 through 31 March 2025. Seasons were combined for this study cohort. Varying season start dates reflect when nirsevimab became available at network primary care practices.

### Study Population

To establish a retrospective primary care attendee cohort of infants eligible for nirsevimab, patients born between 1 April and 30 September during each RSV season with at least 1 primary care visit within 14 days of birth were included. Infants born outside of RSV season (from 1 April through 30 September) are ineligible to receive nirsevimab in the birth hospital and thus instructed to receive it at the start of RSV season in October. Therefore, all children in this primary care attendee cohort were eligible to receive nirsevimab at the start of RSV season in October from their primary care provider (and ineligible to receive nirsevimab in the birth hospital). Children with a history of maternal RSV vaccine administration were excluded. Children’s Hospital of Philadelphia implemented a clinical decision support system alerting clinicians to patients eligible to receive nirsevimab based on age and weight while also checking for contraindications, including maternal RSV vaccination and drug allergy during primary care encounters.

Infants were followed from time zero until the earliest of outcome occurrence or administrative censoring at the end of each RSV season (31 March in 2024 and 2025). To avoid selection bias by conditioning on postbaseline information, we did not include a variable to assess migration out of the healthcare system. Given the short follow-up period (≤6 months) and the expectation that out-migration during infancy is uncommon, infants without recorded outcomes were assumed to remain under observation until the administrative end of follow-up.

### Sequential Target Trial Protocol

To assess the effectiveness of nirsevimab, we designed our observational study cohort to emulate a sequence of target trials comparing nirsevimab with no vaccination to determine its effectiveness in reducing antibiotic prescribing to children with ARTIs. The target trial protocol was developed according to the Transparent Reporting of Observational Studies Emulating a Target Trial (TARGET) guideline that was developed using the Enhancing the Quality and Transparency of Health Research (EQUATOR) framework [16]. Specifications of each component of the target trial and observational emulation are provided in Table 1. Eligibility criteria included being a member of the primary care attendee cohort, no prior receipt of nirsevimab, and no prior receipt of maternal RSV vaccination. Randomized control studies that informed the design of the target trial emulated here include the MELODY Study and HARMONIE Study [17, 18]. Emulating a sequence of target trials allowed for data to be aligned with a clearly defined causal contrast given that a randomized trial evaluating nirsevimab efficacy at reducing antibiotic prescribing may not be practical, feasible, or ethical [19, 20].

**Table 1.** Target Trial Protocol

	Target Trial	Observational Emulation
Eligibility criteria	Infants with established follow-up care within a primary care network	Infants with established follow-up care at a CHOP primary care practice
Treatment strategies	Single intramuscular dose of nirsevimab (50 mg or 100 mg based on body weight) versus placebo	Single intramuscular dose of nirsevimab (50 mg or 100 mg based on body weight) versus no nirsevimab receipt
Assignment procedures	Participants randomized in a 1:1 ratio to receive nirsevimab or placebo	No randomization, eligible participants classified into 2 groups based on nirsevimab receipt from CHOP EHR
Follow-up	Follow-up started immediately after administration of nirsevimab or placebo and followed through 150 d post-dose	Follow-up for each emulated trial began at that trial's baseline and continued until 6 m or until the outcome
Outcome(s)	Primary outcome: (1) outpatient encounters for ARTIs with an antibiotic prescription, (2) outpatient encounters for bronchiolitis with an outpatient encounter with an antibiotic prescription, and (3) RSV-associated hospitalizations with an antibiotic prescription	Primary outcome: (1) outpatient encounters for ARTIs with an antibiotic prescription, (2) outpatient encounters for bronchiolitis with an outpatient encounter with an antibiotic prescription, and (3) RSV-associated hospitalizations with an antibiotic prescription
Causal contrast(s)	Intention-to-treat analysis was used to compare nirsevimab and placebo groups	Intention-to-treat analysis was used to compare nirsevimab and placebo groups
Identifying assumptions	Positivity, consistency, and ignorabilty assumptions hold through successful study design	No unmeasured confounders; no time-varying confounders. Positivity, consistency, and ignorability assumptions hold through covariate adjustments and modeling
Data analysis plan	We obtained the effect of nirsevimab using a Cox proportional hazards model. Cumulative incidence curves were estimated	We compared the time to first outcome event between those who received nirsevimab and those who did not using marginal structural models with adjustment for baseline covariates. Cumulative incidence curves were estimated

Abbreviations: ARTIs, acute respiratory tract infections; CHOP, Children’s Hospital of Philadelphia; EHR, electronic health record; RSV, respiratory syncytial virus.

To emulate the target trial, we implemented a sequential trial design by dividing the study period into 6 monthly intervals. At the start of each sequential trial (time zero), infants who met the eligibility criteria, had not previously received nirsevimab, or experienced the outcome were included. Infants were assigned to the treatment strategy corresponding to whether nirsevimab was administered at time zero according to the medication administration record. Infants who did not receive nirsevimab at time zero were assigned to the nontreatment strategy. Infants who remained eligible could contribute to multiple sequential trials until they experienced the outcome [21, 22]. This design ensures that infants assigned to each treatment strategy share the same time zero and calendar time within the RSV season.

Within each trial, patients were followed up to 6 months or censored at the time of the outcome. There were no differences in ascertaining the outcomes with the observational data than in the target trial. Due to the lack of randomized assignment, identification of causal effects requires the assumption of ignorability; that is, after adjusting for measured potential confounders, treatment assignment is independent of potential outcomes [23, 24].

### Outcomes

Outcomes included (1) outpatient encounters for ARTIs with an antibiotic prescription, (2) outpatient encounters for bronchiolitis with an antibiotic prescription, and (3) RSV-associated hospitalizations with an antibiotic prescription. Each outcome was analyzed separately, and infants were followed from time zero until the first occurrence of the outcome of interest or administrative censoring at the end of follow-up. Given that outcomes were modeled separately, infants could contribute to multiple outcome analyses.

Although nirsevimab specifically prevents only RSV infection, ARTIs and bronchiolitis were chosen as primary outcomes because testing for respiratory viruses (including RSV) is rarely performed on outpatients, and the clinical diagnosis of bronchiolitis alone would exclude nonspecific ARTIs often caused by RSV. All hospitalized patients with respiratory symptoms are tested for a panel of viruses, including RSV.

### Covariates

Baseline variables included practice location, sex, race, ethnicity, preferred language, gestational age, birthweight, and insurance type. Race and ethnicity data were self-reported by patients in the EHR. Although race is a social construct, it is included in the analyses because differences in both nirsevimab receipt and antibiotic prescribing by race have previously been shown [23, 24]. Potential confounders, including sex, race, ethnicity, language, complex chronic conditions (CCCs), and insurance type, were categorical variables, while age at the start of each emulated trial was a continuous variable. Medical complexity was determined using the CCC algorithm (version 3.0) to identify patients with medical conditions that are typically chronic based on groupings of *International Classification of Diseases, Tenth Edition* (*ICD-10*) codes [25]. Respiratory syncytial virus season period was defined based on the month at the start of each emulated trial and categorized as prepeak (October–November), peak (December–January), or postpeak (February–March).

### Statistical Methods

We compared demographic and clinical characteristics between infants who did and did not receive nirsevimab. We estimated causal effects by emulating a sequence of 6 monthly target trials during RSV season. Because continuous-time nirsevimab receipt and outcome dates were discretized into monthly intervals, the implementation effectively included an implicit 1 month grace period for treatment initiation [26]. Eligible trial records not temporally consistent with the initial treatment classification were randomly assigned to 1 of the 2 treatment strategies [26]. The target estimand was the intention-to-treat effect.

We used marginal structural models to estimate cumulative risks under each treatment strategy. The discrete-time hazard of the outcome was modeled using pooled logistic regression with indicators for follow-up intervals. Potential confounding due to nonrandom treatment assignments were addressed by including baseline covariates in the pooled logistic outcome models. Models were adjusted for age at the start of the emulated trial, sex, race, medical complexity, practice membership, and RSV season period. Because infants could contribute observations to multiple sequential trials and follow-up intervals, robust sandwich variance estimators were used to account for weighting and within-infant correlation [21].

The marginal structural models produced (1) estimated cumulative incidence curves, (2) risk differences between nirsevimab and no nirsevimab groups at each follow-up time, and (3) their pointwise 95% CIs. Nirsevimab effectiveness (NE) at 6 months was calculated as NE = 1 − RR, where RR (risk ratio) was derived from the estimated cumulative risks at 6 months obtained from the marginal structural models with 95% CIs approximated using the Delta method [27]. The date of data extraction was 25 July 2025, and study data were collected and managed using Arcus resources hosted by CHOP [28]. All data fields were validated via manual review of 100 randomly selected charts in the EHR. All analyses were conducted using R version 4.3.2., and the TrialEmulation R package was used [20].

### Secondary Analyses

When evaluating the distribution of ARTIs, greater than 90% were attributable to acute otitis media (AOM) or an unspecified viral infection. Therefore, exploratory secondary analyses evaluated outpatient encounters for AOM with an antibiotic prescription and outpatient encounters for viral respiratory infection with an antibiotic prescription. ICD-10 codes and antibiotics used to define outcomes are provided in Supplementary Tables 1–4.

## RESULTS

Of 18 805 patients born between 1 April and 30 September during 2023 and 2024, 16 058 had at least 1 primary care visit within 14 days of birth. After excluding 717 children due to maternal RSV vaccination, 15 341 were considered eligible and available to receive nirsevimab (Figure 1).

**Figure 1.**
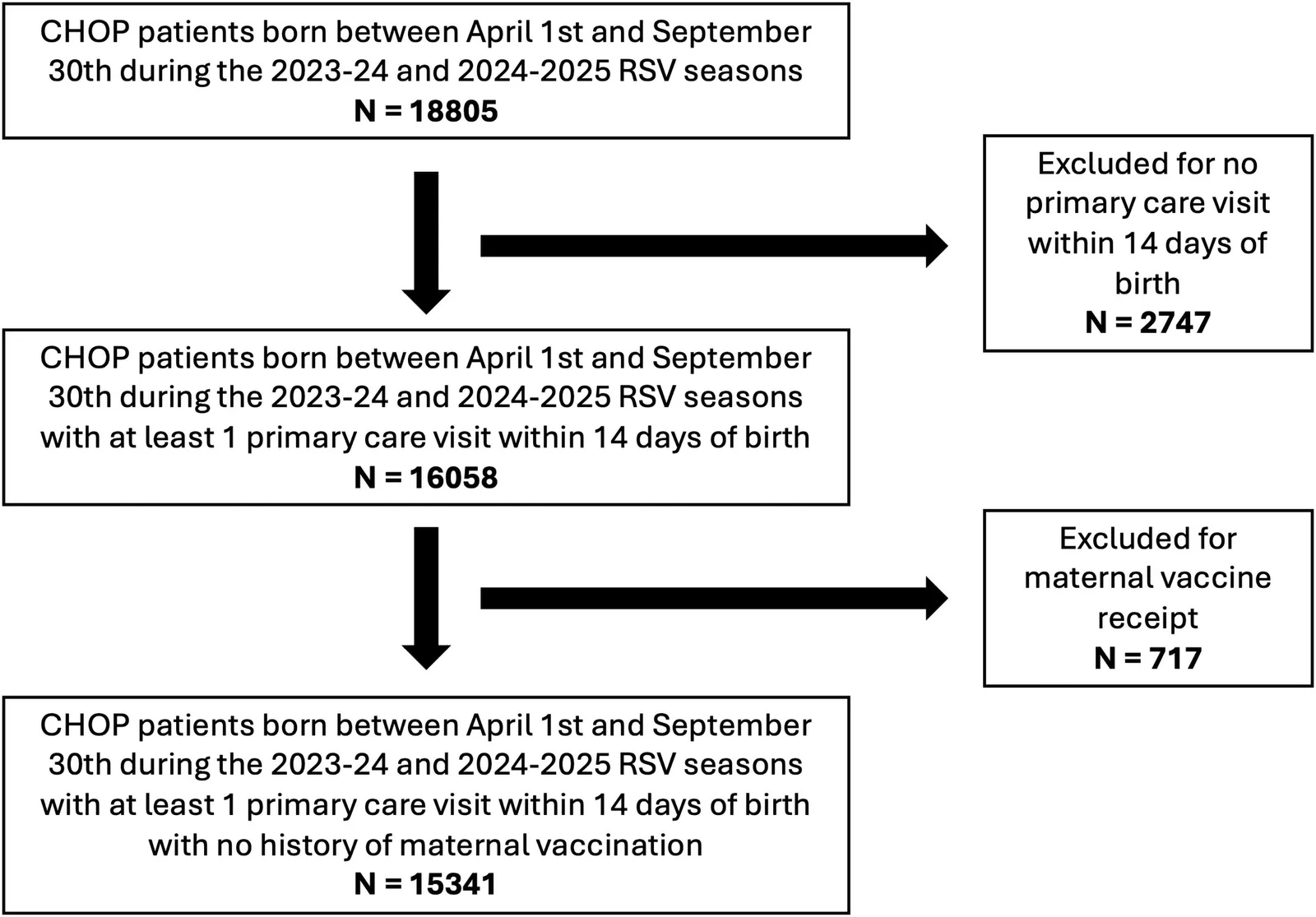
Study flow diagram. Abbreviations: CHOP, Children’s Hospital of Philadelphia; RSV, respiratory syncytial virus.

Patients in this cohort were an average of 3.5 months (SD 1.7) at the start of RSV season, 48.7% were female, 24.2% were Black, and 11.6% were Hispanic/Latino. Overall, 38.0% of patients were publicly insured, and 11.2% had CCCs. The preferred language of most (94.7%) patients in the cohort was English, 5.5% of patients were born preterm, and 5.2% had low birthweight. Additional cohort characteristics are provided in Table 2. Overall, 7413 (48.3%) patients received nirsevimab. Infants who received nirsevimab and those who did not receive nirsevimab resembled each other on most measured covariates (Table 2). However, those who received nirsevimab had a lower average age at the start of RSV season. Chronic conditions characterized by CCCs across treatment groups are provided in Supplementary Table 5.

**Table 2.** Cohort Characteristics

	Did Not Receive Nirsevimab (N = 7928)	Received Nirsevimab (N = 7413)	Overall (N = 15 341)
Sex			
Female	3796 (47.9%)	3677 (49.6%)	7473 (48.7%)
Male	4132 (52.1%)	3736 (50.4%)	7868 (51.3%)
Race			
American Indian or Alaska Native	11 (0.1%)	5 (0.1%)	16 (0.1%)
Asian	370 (4.7%)	432 (5.8%)	802 (5.2%)
Black or African American	2099 (26.5%)	1619 (21.8%)	3718 (24.2%)
Multiple races	616 (7.8%)	594 (8.0%)	1210 (7.9%)
Native Hawaiian or other Pacific Islander	4 (0.1%)	4 (0.1%)	8 (0.1%)
Other/unknown	999 (12.6%)	922 (12.4%)	1921 (12.5%)
White	3829 (48.3%)	3837 (51.8%)	7666 (50.0%)
Ethnicity			
Hispanic or Latino	912 (11.5%)	866 (11.7%)	1778 (11.6%)
Non-Hispanic or non-Latino	6477 (81.7%)	6097 (82.2%)	12 574 (82.0%)
Other/unknown	539 (6.8%)	450 (6.1%)	989 (6.4%)
Language			
English	7527 (94.9%)	7002 (94.5%)	14 529 (94.7%)
Non-English	401 (5.1%)	411 (5.5%)	812 (5.3%)
Age (months)			
Mean (SD)	3.92 (1.70)	3.06 (1.66)	3.50 (1.73)
Median [min, max]	4.00 [1.00, 7.00]	3.00 [1.00, 7.00]	3.00 [1.00, 7.00]
Birth weight			
Low (<2.5 kg)	380 (4.8%)	420 (5.7%)	800 (5.2%)
Normal (≥2.5 kg)	7534 (95.0%)	6983 (94.2%)	14 517 (94.6%)
Missing	14 (0.2%)	10 (0.1%)	24 (0.2%)
Gestational age			
Preterm (<37 wks)	399 (5.0%)	448 (6.0%)	847 (5.5%)
Term (≥37 wks)	7209 (90.9%)	6694 (90.3%)	13 903 (90.6%)
Missing	320 (4.0%)	271 (3.7%)	591 (3.9%)
Complex chronic condition (CCC)			
No	7015 (88.5%)	6610 (89.2%)	13 625 (88.8%)
Yes	913 (11.5%)	803 (10.8%)	1716 (11.2%)
Insurance			
Private	4664 (58.8%)	4792 (64.6%)	9456 (61.6%)
Public	3215 (40.6%)	2616 (35.3%)	5831 (38.0%)
Self-pay	49 (0.6%)	5 (0.1%)	54 (0.4%)
Child Opportunity Index			
Very high	2774 (35.0%)	2707 (36.5%)	5481 (35.7%)
High	1443 (18.2%)	1232 (16.6%)	2675 (17.4%)
Moderate	761 (9.6%)	561 (7.6%)	1322 (8.6%)
Low	795 (10.0%)	689 (9.3%)	1484 (9.7%)
Very low	1203 (15.2%)	997 (13.4%)	2200 (14.3%)
Missing	952 (12.0%)	1227 (16.6%)	2179 (14.2%)

Abbreviations: max, maximum; min, minimum; SD, standard deviation.

The estimated NE at 6 months of follow-up for preventing antibiotic prescribing for ARTIs was 14.4% (95% CI: 7.8%, 20.6%). The estimated NE for preventing antibiotic prescribing for bronchiolitis was 40.3% (95% CI: 25.1%, 52.5%). The NE for preventing antibiotic prescribing for RSV-related hospitalizations was 69.4% (95% CI: 4.8%, 90.1%). Cumulative incidence curves for each outcome are shown in Figure 2, and risk differences for each outcome are provided in Table 3.

**Figure 2.**
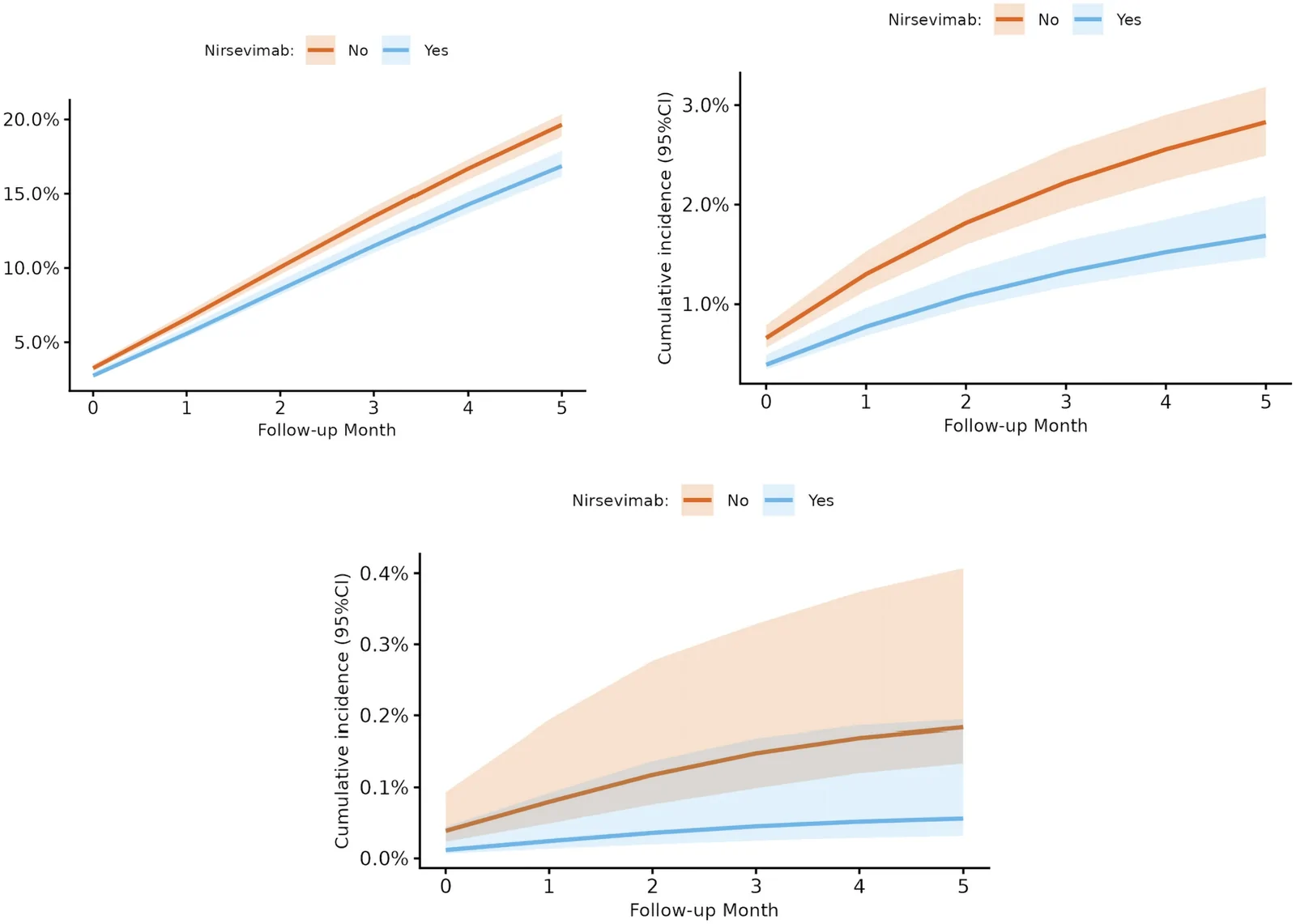
Cumulative incidence of outcomes. Top left: cumulative incidence of first antibiotic prescriptions for outpatient acute respiratory tract infection (ARTI) encounters. Top right: cumulative incidence of first antibiotic prescriptions for outpatient bronchiolitis encounters. Bottom: cumulative incidence of first antibiotic prescriptions for respiratory syncytial virus (RSV)-related hospitalizations. Predicted cumulative incidence of outcomes for infants receiving nirsevimab and those not receiving nirsevimab. Shaded areas represent pointwise 95% CIs derived from the marginal structural models. Abbreviation: CI, confidence interval.

**Table 3.** Nirsevimab Effectiveness Against Antibiotics Prescribed for Study Outcomes at 6 Months

	Number of Patients At Risk		Risk Difference (95% CI)	Nirsevimab Effectiveness (95% CI)
	Vaccinated Group	Unvaccinated Group		
Antibiotics prescribed for outpatient encounters for ARTIs	6116	6320	0.028 (.018, .035)	0.14 (.08, .21)
Antibiotics prescribed for outpatient encounters for bronchiolitis	7284	7681	0.011 (.007, .014	0.40 (.25, .53)
Antibiotics prescribed for RSV-related hospitalizations	7910	7409	0.001 (.0004, .003)	0.69 (.05, .90)
Antibiotics prescribed for outpatient encounters for AOM	6207	6421	0.051 (.043, .057)	0.27 (.21, .32)
Antibiotics prescribed for outpatient encounters for viral respiratory infections	6894	7195	0.020 (.012, .025)	0.23 (.13, .31)

Abbreviations: AOM, acute otitis media; ARTIs, acute respiratory tract infections; CI, confidence interval; RSV, respiratory syncytial virus.

In our exploratory secondary analysis, the estimated NE for preventing antibiotic prescribing was 26.8% (95% CI: 20.8%, 32.4%) and 22.5% (95% CI: 12.6%, 31.3%) for AOM and viral respiratory infections, respectively. Risk differences for each outcome are provided in Table 3.

## DISCUSSION

This emulated target trial of infants within a large, diverse pediatric care network found that nirsevimab administration decreased antibiotic use for respiratory infections across outpatient and inpatient settings among infants during their first RSV season. Although the primary purpose of nirsevimab is to prevent RSV-associated hospitalizations, this study demonstrates a clinically meaningful reduction in RSV-associated antibiotic use, revealing the additional impact of nirsevimab as an important tool for antibiotic stewardship.

While randomized controlled trials have demonstrated the effectiveness of nirsevimab at preventing RSV-associated hospitalizations and RSV-associated lower respiratory tract infections, potential reduction in antibiotic use was not assessed [17, 29]. A pooled secondary analysis combining infants who participated in a Phase 2B and Phase 3 MELODY trial reported that infants who received nirsevimab were 24% less likely to be prescribed antibiotics for any indication compared to infants who received placebo [30]. Our study, conducted after FDA approval and primary care distribution of nirsevimab across a pediatric care network serving a diverse cohort of children in a wide range of practice settings, demonstrates comparable nirsevimab effectiveness in areal-world setting.

Our observation is analogous to prior studies of influenza and pneumococcal vaccines, which were associated with reduced antibiotic prescribing. The mechanisms for these effects are likely similar, including the prevention of viral illnesses that can mimic bacterial infections (eg influenza and RSV) as well as prevention of primary bacterial infections or bacterial superinfections (eg, pneumococcal pneumonia and RSV-associated AOM), all of which can lead to healthcare encounters during which antibiotics may be prescribed. This is consistent with a recent report that nirsevimab receipt led to a significantly decreased rate of AOM [31]. The magnitude of reduction in antibiotic prescriptions observed in the present study (22.5%–26.8%) is comparable to reported estimates for the influenza and pneumococcal vaccines (10%–40%) [12, 32–35].

While the primary goal of nirsevimab is to reduce RSV-associated hospitalizations and outpatient encounters for RSV, less frequent antibiotic use has the added effects of avoiding adverse drug effects, protecting the infant microbiome, and reducing the development of antibiotic resistance [28, 36]. Inappropriate and unnecessary antibiotic use within the first year of life and gut microbiome disruption has been associated with a variety of chronic conditions, including atopic dermatitis, asthma, autoimmune disease, and obesity [28, 37]. Our findings suggest that nirsevimab has a dual impact in preventing RSV-associated hospitalizations and reducing antibiotic prescribing in both inpatient and outpatient settings.

Our study has multiple strengths. The cohort is large and diverse, including academic and community practices serving children with a range of racial, ethnic, and socioeconomic backgrounds across urban and suburban settings. All practices use a shared, comprehensive EHR and refer the majority of children to 2 emergency departments, 4 urgent care centers, and 2 inpatient facilities (all also sharing the same EHR). Although retrospective in design, the use of target trial emulation helped mitigate biases that are common in other observational studies. Our study cohort evaluated outcomes across 2 consecutive RSV seasons, allowing assessment of both the effectiveness of nirsevimab and consistency of its protection.

Our study has several limitations. Because this study exclusively evaluated a pediatric care network across Pennsylvania and New Jersey, our findings may not be nationally generalizable. Because of its retrospective design, this study was limited by data recorded in the EHR and, therefore, is subject to potential misclassification of exposures (ie, nirsevimab administration, although this is highly unlikely because nirsevimab was only available at primary care sites during the period under study) and outcomes (eg, the uncommon use of outside hospitals and urgent care centers leading to antibiotic use unrecorded in the network EHR). We also could not account for potential unmeasured confounders, such as healthcare utilization, which might be related to both the likelihood of nirsevimab receipt and the likelihood of having a medically attended encounter (although this might have, if anything, attenuated the observed impact of nirsevimab on antibiotic exposure). Maternal vaccine receipt was not validated using external maternal health records and was self-reported during primary care encounters.

## CONCLUSIONS

Nirsevimab administration reduced antibiotic use for ARTIs across settings, highlighting its secondary importance as an effective tool for combating antibiotic overuse. This decrease in antibiotic use appears to be driven largely by a reduction in antibiotic prescribing for both viral infections and AOM. Increasing currently suboptimal rates of nirsevimab coverage among infants in their first year of life should be a priority not only to prevent the morbidity associated with RSV bronchiolitis but also to supplement existing antibiotic stewardship efforts.

## Supplementary Material

ciag406_Supplementary_Data

## Supplementary Data

Supplementary materials are available at *Clinical Infectious Diseases* online. Consisting of data provided by the authors to benefit the reader, the posted materials are not copyedited and are the sole responsibility of the authors, so questions or comments should be addressed to the corresponding author.
