# Supplementary material for: The Effectiveness of Nirsevimab on Antibiotic Use in Children Using Target Trial Emulation
Source: Clin Infect Dis. 2026 Aug 6;83(3):e487–94. doi: 10.1093/cid/ciag406 (PMC13644860; doi:10.1093/cid/ciag406)
Supplement: Supplementary file 1 [file ciag406_supplementary_data.docx]

**Supplemental Table 1. ARTI ICD-10 codes**

| ICD-10 Code | Diagnosis |
| --- | --- |
| A37 | Whooping cough |
| A37.0 | Whooping cough due to Bordetella pertussis |
| A37.1 | Whooping cough due to Bordetella pertussis |
| A37.8 | Whooping cough due to other Bordetella species |
| A37.9 | Whooping cough, unspecified species |
| A38 | Scarlet fever |
| A38.0 | Scarlet fever with otitis media |
| A48.1 | Legionnaire’s disease |
| H10 | Conjunctivitis |
| H10.0 | Mucopurulent conjunctivitis |
| H10.1 | Acute atopic conjunctivitis |
| H10.2 | Other acute conjunctivitis |
| H10.3 | Unspecified acute conjunctivitis |
| H10.4 | Chronic conjunctivitis |
| H10.8 | Other conjunctivitis |
| H10.9 | Unspecified conjunctivitis |
| H66 | Suppurative and unspecified otitis media |
| H66.0 | Acute suppurative otitis media |
| H66.00 | Acute suppurative otitis media without spontaneous rupture of ear drum |
| H66.001 | Acute suppurative otitis media without spontaneous rupture of ear drum, right ear |
| H66.002 | Acute suppurative otitis media without spontaneous rupture of ear drum, left ear |
| H66.003 | Acute suppurative otitis media without spontaneous rupture of ear drum, bilateral |
| H66.004 | Acute suppurative otitis media without spontaneous rupture of ear drum, recurrent, right ear |
| H66.005 | Acute suppurative otitis media without spontaneous rupture of ear drum, recurrent, left ear |
| H66.006 | Acute suppurative otitis media without spontaneous rupture of ear drum, recurrent, bilateral |
| H66.009 | Acute suppurative otitis media without spontaneous rupture of ear drum, unspecified ear |
| H66.01 | Acute suppurative otitis media with spontaneous rupture of ear drum |
| ICD-10 Code | Diagnosis |
| H66.011 | Acute suppurative otitis media with spontaneous rupture of ear drum, right ear |
| H66.012 | Acute suppurative otitis media with spontaneous rupture of ear drum, left ear |
| H66.013 | Acute suppurative otitis media with spontaneous rupture of ear drum, bilateral |
| H66.015 | Acute suppurative otitis media with spontaneous rupture of ear drum, recurrent, right ear |
| H66.019 | Acute suppurative otitis media with spontaneous rupture of ear drum, unspecified ear |
| H66.1 | Chronic tubotympanic suppurative otitis media |
| H66.10 | Chronic tubotympanic suppurative otitis media – unspecified |
| H66.11 | Chronic tubotympanic suppurative otitis media, right ear |
| H66.12 | Chronic tubotympanic suppurative otitis media, left ear |
| H66.13 | Chronic tubotympanic suppurative otitis media, bilateral |
| H66.2 | Chronic atticoantral suppurative otitis media |
| H66.20 | Chronic atticoantral suppurative otitis media, unspecified ear |
| H66.21 | Chronic atticoantral suppurative otitis media, right ear |
| H66.22 | Chronic atticoantral suppurative otitis media, left ear |
| H66.23 | Chronic atticoantral suppurative otitis media, bilateral |
| H66.3 | Other chronic suppurative otitis media |
| H66.4 | Suppurative otitis media – unspecified |
| H66.43 | Chronic suppurative otitis media, unspecified ear |
| H66.9 | Otitis media – unspecified |
| H66.90 | Otitis media, unspecified, unspecified ear |
| H66.91 | Otitis media, unspecified, right ear |
| H66.92 | Otitis media, unspecified, left ear |
| H66.93 | Otitis media, unspecified, bilateral |
| H67 | Otitis media in diseases classified elsewhere |
| H67.1 | Otitis media in diseases classified elsewhere, right ear |
| H67.2 | Otitis media in diseases classified elsewhere, left ear |
| H67.3 | Otitis media in diseases classified elsewhere, bilateral |
| H67.9 | Otitis media in diseases classified elsewhere, unspecified ear |
| J00 | Acute nasopharyngitis (common cold) |
| J01 | Acute sinusitis |
| ICD-10 Code | Diagnosis |
| J01.0 | Acute maxillary sinusitis |
| J01.1 | Acute frontal sinusitis |
| J01.2 | Acute ethmoidal sinusitis |
| J01.3 | Acute sphenoidal sinusitis |
| J01.4 | Acute pansinusitis |
| J01.8 | Other acute sinusitis |
| J01.9 | Acute sinusitis, unspecified |
| J02 | Acute pharyngitis |
| J02.0 | Streptococcal pharyngitis |
| J02.8 | Acute pharyngitis due to other specified organisms |
| J02.9 | Acute pharyngitis, unspecified |
| J03 | Acute tonsillitis |
| J03.0 | Streptococcal tonsillitis |
| J03.8 | Acute tonsillitis due to other specified organisms |
| J03.9 | Acute tonsillitis – unspecified |
| J06 | Acute upper respiratory infections of multiple and unspecified sites |
| J06.0 | Acute laryngopharyngitis |
| J06.9 | Acute upper respiratory infection – unspecified |
| J13 | Pneumonia due to Streptococcus pneumoniae |
| J14 | Pneumonia due to Haemophilus influenzae |
| J15 | Bacterial pneumonia, not elsewhere classified |
| J15.0 | Pneumonia due to Klebsiella pneumoniae |
| J15.1 | Pneumonia due to Pseudomonas |
| J15.2 | Pneumonia due to staphylococcus |
| J15.20 | Pneumonia due to staphylococcus, unspecified |
| J15.21 | Pneumonia due to S. aureus |
| J15.29 | Pneumonia due to other staphylococcus |
| J15.3 | Pneumonia due to streptococcus, group B |
| J15.4 | Pneumonia due to other streptococci |
| J15.5 | Pneumonia due to E. coli |
| ICD-10 Code | Diagnosis |
| J15.6 | Pneumonia due to other gram-negative bacteria |
| J15.7 | Pneumonia due to Mycoplasma pneumoniae |
| J15.8 | Pneumonia due to other specified bacteria |
| J15.9 | Unspecified bacterial pneumonia |
| J16.8 | Pneumonia due to other specified infectious organisms |
| J17 | Pneumonia in diseases classified elsewhere |
| J18 | Pneumonia due to unspecified organism |
| J18.0 | Bronchopneumonia due to unspecified organism |
| J18.1 | Lobar pneumonia due to unspecified organism |
| J18.2 | Hypostatic pneumonia due to unspecified organism |
| J18.8 | Other pneumonia due to unspecified organism |
| J18.9 | Pneumonia due to unspecified organism |
| J18.9 | Pneumonia, unspecified organism |
| J20 | Acute bronchitis |
| J20.0 | Acute bronchitis due to Mycoplasma pneumoniae |
| J20.1 | Acute bronchitis due to Haemophilus influenzae |
| J20.2 | Acute bronchitis due to streptococcus |
| J20.3 | Acute bronchitis due to coxsackievirus |
| J20.4 | Acute bronchitis due to parainfluenza |
| J20.5 | Acute bronchitis due to RSV |
| J20.6 | Acute bronchitis due to rhinovirus |
| J20.7 | Acute bronchitis due to echovirus |
| J20.8 | Acute bronchitis due to other specified organisms |
| J20.9 | Acute bronchitis, unspecified |
| J21 | Acute bronchiolitis |
| J21.0 | Acute bronchiolitis due to RSV |
| J21.1 | Acute bronchiolitis due to human metapneumovirus |
| J21.1 | Acute bronchiolitis due to human metapneumovirus |
| J21.8 | Acute bronchiolitis due to other specified organisms |
| J21.9 | Acute bronchiolitis, unspecified |
| ICD-10 Code | Diagnosis |
| J22 | Unspecified acute lower respiratory infection |
| J40 | Bronchitis, not specified as acute or chronic |

ICD-10 codes used for defining AOM are highlighted in green; ICD-10 codes used for defining viral respiratory infections are highlighted in blue

**Supplemental Table 2. Bronchiolitis ICD-10 codes**

| ICD-10 Code | Diagnosis |
| --- | --- |
| B97.4 | RSV as the cause of diseases classified elsewhere |
| J12.1 | RSV pneumonia |
| J20.5 | Acute bronchitis due to RSV |
| J21.0 | Acute bronchiolitis due to RSV |
| J21.1 | Acute bronchiolitis due to human metapneumovirus |
| J21.8 | Acute bronchiolitis due to other specific organisms |
| J21.9 | Acute bronchiolitis – unspecified |

**Supplemental Table 3. RSV-associated hospitalization ICD-10 codes**

| ICD-10 Code | Diagnosis |
| --- | --- |
| B97.4 | RSV as the cause of diseases classified elsewhere |
| J12.1 | RSV pneumonia |
| J20.5 | Acute bronchitis due to RSV |
| J21.0 | Acute bronchiolitis due to RSV |

**Supplemental Table 4. List of selected antibiotics**

| Penicillin | Clindamycin |
| --- | --- |
| Ampicillin | Erythromycin |
| Ciprofloxacin | Clarithromycin |
| Levofloxacin | Doxycycline |
| Moxifloxacin | Tetracycline |
| Ceftriaxone | Minocycline |
| Cefdinir | Amoxicillin-clavulanate |
| Cephalexin | Sulfamethoxazole |
| Cephalosporin | Azithromycin |
| TMP-SMX | Cefixime |

**Supplemental Table 5. Distribution of CCCs Across Study Cohort.**

|  | **Did Not Receive Nirsevimab** **(N=7928)** | **Received Nirsevimab** **(N=7413)** | **Overall** **(N=15341)** |
| --- | --- | --- | --- |
| **Cardiovascular** | 81 (1.0%) | 84 (1.1%) | 165 (1.1%) |
| **Gastrointestinal** | 13 (0.2%) | 19 (0.3%) | 32 (0.2%) |
| **Hematologic or Immunologic** | 247 (3.1%) | 220 (3.0%) | 467 (3.0%) |
| **Malignancy** | 11 (0.1%) | 8 (0.1%) | 19 (0.1%) |
| **Metabolic** | 196 (2.5%) | 186 (2.5%) | 382 (2.5%) |
| **Neonatal** | 22 (0.3%) | 25 (0.3%) | 47 (0.3%) |
| **Congenital or Genetic** | 235 (3.0%) | 147 (2.0%) | 382 (2.5%) |
| **Renal** | 52 (0.7%) | 54 (0.7%) | 106 (0.7%) |
| **Respiratory** | 130 (1.6%) | 134 (1.8%) | 264 (1.7%) |
| **Neurologic or Neuromuscular** | 2 (0.0%) | 10 (0.1%) | 12 (0.1%) |
